# Perfluorooctanoic acid (PFOA) exposure promotes proliferation, migration and invasion potential in human breast epithelial cells

**DOI:** 10.1007/s00204-018-2181-4

**Published:** 2018-03-03

**Authors:** Paula Pierozan, Fredrik Jerneren, Oskar Karlsson

**Affiliations:** Department of Pharmaceutical Biosciences, Uppsala University, Box 591, 751 24 Uppsala, Sweden

**Keywords:** Endocrine disrupting chemicals, EDCs, MCF-10A cells, Breast cancer, Cyclin D, P27

## Abstract

Despite significant advances in early detection and treatment, breast cancer remains a major cause of morbidity and mortality. Perfluorooctanoic acid (PFOA) is a suspected endocrine disruptor and a common environmental pollutant associated with various diseases including cancer. However, the effects of PFOA and its mechanisms of action on hormone-responsive cells remain unclear. Here, we explored the potential tumorigenic activity of PFOA (100 nM–1 mM) in human breast epithelial cells (MCF-10A). MCF-10A cells exposed to 50 and 100 µM PFOA demonstrated a higher growth rate compared to controls. The compound promoted MCF-10A proliferation by accelerating *G*_0_/*G*_1_ to *S* phase transition of the cell cycle. PFOA increased cyclin D1 and CDK4/6 levels, concomitant with a decrease in p27. In contrast to previous studies of perfluorooctane sulfate (PFOS), the estrogen receptor antagonist ICI 182,780 had no effect on PFOA-induced cell proliferation, whereas the PPARα antagonist GW 6471 was able to prevent the MCF-10A proliferation, indicating that the underlying mechanisms involve PPARα-dependent pathways. Interestingly, we also showed that PFOA is able to stimulate cell migration and invasion, demonstrating its potential to induce neoplastic transformation of human breast epithelial cells. These results suggest that more attention should be paid to the roles of PFOA in the development and progression of breast cancer.

## Introduction

Breast cancer is one of the most common malignancies that women in Western countries may develop in their lifetime (Gullick et al. 1998). During the last decades, the breast cancer incidence has globally increased and accounts for over 20% of the total cancer cases and about 14% of cancer deaths among females (Jemal et al. 2011). Despite extensive research efforts to understand and treat breast cancer, risk factors and cellular processes that can lead to the onset of mammary carcinogenesis have yet to be elucidated in detail. About 5–10% of the cases are due to inheritance of risk genes such as BRCA1 and BRCA2 (Campeau et al. 2008). Several common persistent organic pollutants are endocrine disrupters and proposed to play an important role in cancer etiology. These compounds have been linked to effects relevant for the development of breast cancer, including tumor promoting, and immunosuppressive activities (Bonefeld-Jorgensen et al. 2011; Kortenkamp 2006). Among them, both estrogenic and non-estrogenic endocrine disruptors are considered to play critical roles in human breast carcinogenesis (Brody et al. 2007). Hence, studies of hormonally active compounds contaminating the environment, food and water are a priority.

In recent years, human and wildlife monitoring studies have ubiquitously detected manmade perfluoroalkyl acids (PFAAs) throughout the world. These chemicals are frequently used in industrial and consumer products, because of their stain-resistant and water-repellant characteristics. The two most widely known PFAAs are perfluorooctanoic acid (PFOA), and perfluorooctane sulfate (PFOS) (Lau et al. 2007). PFOA has been used in products such as non-stick cookware, cosmetics, upholstery, and is known to bio-accumulate in food chains (Bartell et al. 2010). In addition, PFOA does not break down in the environment and can be found in drinking water at concentrations ranging from picograms to micrograms per liter (Rayne and Forest 2009; Shin et al. 2011). PFOA has also been detected in blood of most people examined in a number of industrialized countries. The exposure levels vary among populations and over time (Bartell et al. 2010; Herrick et al. 2017). In blood, PFOA has been reported to reach values between 7 and 9370 ng/mL, whereas the median level for the general US population is considered to be approximately 5 ng/mL (Emmett et al. 2006; Steenland et al. 2010). The compound is not metabolized in the body, and the human half-life is estimated between 4 and 5 years (Emmett et al. 2006; Lau et al. 2007). According with this, a study using post-mortem samples found concentrations around 3.8 and 1.4 ng/g in liver and adipose tissue, respectively (Maestri et al. 2006).

PFOA exposure may lead to a variety of adverse effects, including hepatotoxicity, immunotoxicity, and developmental toxicity (Lau et al. 2007; Yang et al. 2000). Moreover, toxicologic data show that dietary intake of PFOA induces tumors of various tissues in rodents (Biegel et al. 2001). Animal studies have also indicated that PFOA exposure affects mammary gland development and increases mammary fibroadenomas (White et al. 2007). Recent epidemiological studies support a possible association between both PFOS and PFOA and tumorigenesis, including liver, pancreatic, and testicular cancer (Barry et al. 2013; Lau et al. 2007; Vieira et al. 2013), as well as breast cancer (Bonefeld-Jorgensen et al. 2011; Wielsoe et al. 2017). PFOS and PFOA are suspected to be endocrine disruptors with estrogenic activity, but the potential for these environmental contaminants to induce estrogenic disruption in mammals is not fully known (Jensen and Leffers 2008; Sonthithai et al. 2016).

Mechanisms by which PFAAs may affect breast tissue can be studied in animal and cell models. MCF-10A is a subline of spontaneously immortalized human breast epithelial cells—derived from human fibrocystic mammary tissue—with characteristics of normal breast epithelium (Soule et al. 1990). These features make the MCF-10A a valuable in vitro model for studies of normal breast cell function and determine the potential of environmental contaminants to induce tumor transformation. We have recently demonstrated that PFOS is capable of transforming MCF-10A to a malignant profile by altering important cell cycle proteins as well as inducing cell migration and invasion (Pierozan and Karlsson 2018).

In this study, we evaluate the potential tumorigenic activity of PFOA in MCF-10A cells. Cell viability, cell counting, and cell cycle analysis were applied to investigate the effects of PFOA exposure on cell proliferation. To better understand the response of MCF-10A cells to PFOA exposure, we studied the regulatory cell cycle proteins, cell migration/invasion and estrogen receptor (ER), pregnane X receptor (PXR) and peroxisome proliferator-activated receptor alpha (PPARα) activation. The findings of this study will help clarify the mechanisms underlying possible effects of PFOA on breast cancer development.

## Materials and methods

### Chemicals

Dimethyl sulfoxide (DMSO), paraformaldehyde, 4′,6-diamidino-2-phenylindole dihydrochloride (DAPI), Triton X-100, propidium iodide (PI), DNAse-free RNAse A, perfluorooctanoic acid, cholera toxin (CT), insulin, 3-(4,5-dimethyl-2-yl)2,5-diphenyl-2H-tetrazolium bromide (MTT), epidermal growth factor (EGF), hydrocortisone and sulforaphane were obtained from Sigma-Aldrich (St Louis, MO, USA). Horse serum, penicillin–streptomycin (P/S), Dulbecco’s Phosphate-Buffered Saline (PBS), Dulbecco’s Modified Eagle’s Medium (DMEM), trypsin solution (0.05%) were obtained from Gibco (Invitrogen, Paisley, UK). p53 monoclonal (DO-7), CDK6 monoclonal (75B9), CDK4 monoclonal (DCS-31) and p21 monoclonal (R.229.6) antibodies were obtained from ThermoFisher Scientific (Rockford, IL, USA). P27 Kip1 (D69C12) and cyclin D1 (92G2) antibodies were obtained from Cell Signaling (Danvers, MA, USA). The secondary antibodies Alexa-Fluor 555 goat anti-mouse IgG or 488 goat anti-rabbit IgG, and the blocking agent (normal goat serum) were obtained from Molecular Probes, Invitrogen. Matrigel Basement Membrane Matrix was obtained from Corning (New York, NY, USA). ER α (sc-8002) and ERβ (sc-8974) monoclonal antibodies were obtained from Santa Cruz Biotechnology (Bergheimer, HD, DE). ICI 182,780, GW 6471 was obtained from Tocris Bioscience (Avonmouth, Bristol, UK).

### Cell culture

MCF-10A cells were obtained from the American Type Culture Collection (ATCC, Manassas, VA, USA). Cells were maintained as a monolayer in 10-cm^2^ tissue culture plastic flasks containing 10 mL of growth medium, trypsinized (0.05%) and split 1:5 every 3 days. Complete growth medium consisted of Dulbecco’s Modifies Eagle Medium with F-12 (DMEM/F-12; GIBCO, Invitrogen, Paisley, UK) supplemented with horse serum (5%), EGF (20 ng/mL), hydrocortisone (0.5 mg/mL), CT (100 ng/mL), insulin (10 mg/mL), and 5 mL P/S. Cell cultures were maintained at 37 °C and 5% CO_2_ in a humidified incubator.

### Exposure of MCF-10A cells to PFOA

MCF-10A cells were trypsinized and resuspended in growth medium, plated in 96-well tissue culture plates (2 × 10^4^ cells/well), and allowed to attach for 24 h in a 5% CO_2_ humidified incubator at 37 °C. After 24 h, the cells were treated with different PFOA concentrations (0–1 mM) dissolved in water and assay medium (growth medium without horse serum and EGF). The cells were incubated for 24, 48 and 72 h. All experiments were repeated three times.

### MTT cell viability assay

MCF-10A cells were treated with different concentrations of PFOA (0–1 mM) for 24, 48 and 72 h, using eight wells for each treatment and three independent experiments. Cell viability was measured by the MTT assay. In brief, 0.5 mg MTT was added to each well of the 96-well plates (containing 100 µL medium and cells) 1 h before the end of incubation with PFOA. The supernatant was then removed and 100 µL DMSO was added to each well, followed by incubation and shaking for 10 min. The formazan product generated during the incubation was solubilized in DMSO and measured at 490 and 630 nm using a Polarstar Optima microplate reader (Bmg Labtech, Offenburg, Germany).

To investigate the participation of receptors on the PFOA effects, the cells were preincubated with the ER receptor blocker ICI 182,780 (100 nM) (Wrobel and Gregoraszczuk 2014), the PXR antagonist sulforaphane (1 µM) (Zhou et al. 2008) and the PPARα blocker GW6471 (1 µM) (Hah et al. 2014) for 30 min, before treatment with PFOA 100 µM for 72 h, and the MTT assay.

### Cell counting by DAPI staining

MCF-10A cells were treated with different concentrations of PFOA (0–1 mM) for 24, 48 and 72 h, with three replicates of each treatment. After the treatment, cells were fixed with 4% paraformaldehyde for 30 min and permeabilized with 0.1% Triton X-100 in PBS for 5 min at room temperature. Cells were stained with DAPI (0.25 mg/mL) for 10 min at room temperature followed by two washes with PBS. Cells were viewed in an ImageXpress Micro XLS Widefield High-Content Analysis System (Molecular Devices, Sunnyvale CA, USA), and images analyzed with the SoftMax Pro Software after digital acquisition (Molecular Devices, Sunnyvale CA, USA).

### Analysis of cell cycle phases and proteins involved in cell cycle regulation

Cells were treated for 24, 48 and 72 h with 100 µM PFOA and processed for PI staining and flow cytometry as described previously (Pozarowski and Darzynkiewicz 2004). Forward and light scatter data were collected in a linear mode. Fluorescence data for 10,000 cells per sample were collected in the FL3 channel on a linear scale. Side- and forward-light scatter parameters were used to identify the cell events and doublets were excluded using gating. Samples were analyzed using a Cytoflex flow cytometer (Beckman Coulter Ltd., Brea, CA, USA). Cells in different cell cycle phases were presented as a percentage of the total number of cells counted.

To evaluate the effects on proteins involved in cell cycle regulation, cells were incubated with anti-cyclin D, anti-CDK4/6, anti-p21/27/53 antibodies and analyzed by flow cytometry using a Cytoflex flow cytometer (Beckman Coulter Ltd., Brea, CA, USA) as described previously (Pierozan and Karlsson 2018). Side and forward scatter of aggregates or lysed cells were determined using log scale SSC/FSC plots with thresholds. Voltage settings for the SSC, FSC and the fluorescent filters were kept constant for all experiments described. The results are expressed as mean fluorescence intensity compared with the controls (% of controls) for 10,000 cells per sample.

### Immunocytochemistry

MCF-10A cells were treated with 100 µM PFOA 72 h and immunocytochemistry was performed as previously described (Pierozan et al. 2016). Negative control reactions were performed by omitting the primary antibody with no observed fluorescence. Cells were examined in an Olympus inverted microscope BX53 (Olympus, Tokyo, Japan), and images were collected with a 20 × objective using constant intensity settings and exposure time for all samples. The intensity of the cell fluorescence was measured using the image J software (ImageJ2), and the fluorescence intensity was estimated as the difference between the measured fluorescence of the cells and the background.

### Migration and invasion assay

Transwell migration and invasion assays were conducted in 96-well plates with 8 µm pore-size membrane inserts (Corning, New York, NY, USA). Cells were treated with 100 µM PFOA for 72 h. After that, 5 × 10^5^ cells were resuspended in 50 µL of assay medium and seeded in the upper chamber of transwells with (invasion assay) or without (migration assay) Matrigel Matrix (200 µg/mL). The lower chamber contained 100 µL growth medium. Cells were incubated for 24 h at 37 °C in a humidified atmosphere with 5% CO_2_. At the end of incubation, noninvasive cells in the upper chamber were removed and invasive cells in the bottom were fixed with 4% formaldehyde and stained with DAPI and counted as described above using an ImageXpress Micro XLS Widefield High-Content Analysis System (Molecular Devices, Sunnyvale, CA, USA), and the SoftMax Pro Software (Molecular Devices, Sunnyvale, CA, USA).

### Western blot analysis

For evaluation of ER protein levels, cells were exposed to 100 µM PFOA or 10 nM 17β-estradiol (E2; positive control) for 72 h. Cells were then washed with ice-cold PBS and lysed with Laemmli lysis buffer. The protein concentration in cell lysates was determined using bicinchoninic acid protein assay (Smith et al. 1985). Equal amounts of protein (30 µg) were separated by sodium dodecyl sulfate–polyacrylamide gel electrophoresis (SDS-PAGE) on 10% gel and transferred (Mini Trans-Blot Electrophoretic Transfer Cell; Bio-Rad, Hercules, CA, USA) to polyvinylidene difluoride membranes for 1 h at 100 V in transfer buffer (25 mM Tris, 192 mM glycine, 20% methanol and 0.1% SDS). The blot was then washed for 20 min in Tris-buffered saline (TBS; 500 mM NaCl, 20 mM Trizma, pH 7.5), followed by 2 h incubation in blocking solution (TBS plus 5% defatted dry milk). After the incubation, the blot was washed twice for 5 min with blocking solution containing 0.05% Tween-20 (T-TBS) and incubated overnight at 4 °C in blocking solution containing monoclonal antibodies diluted 1:1000. The blot was then washed twice for 5 min with T-TBS and incubated for 2 h in a solution containing peroxidase-conjugated rabbit anti-mouse IgG diluted 1:2000 or peroxidase-conjugated anti-rabbit IgG diluted 1:2000. The blot was washed twice with T-TBS for 5 min and twice with TBS for 5 min. The blot was developed with the chemiluminescence ECL kit (Little Chalfont, UK).

### Statistical analysis

The results were presented as means ± standard deviation (SD) for each experimental group of at least three individual samples. Differences between the groups were analyzed by one-way analysis of variance (ANOVA) followed by Tukey–Kramer multiple test, or by Student’s *t* test when comparing only two groups, using Graphpad Prism 7 software.

## Results

### PFOA-induced cell death and proliferation are dependent on the time and concentration

**Fig. 1:**
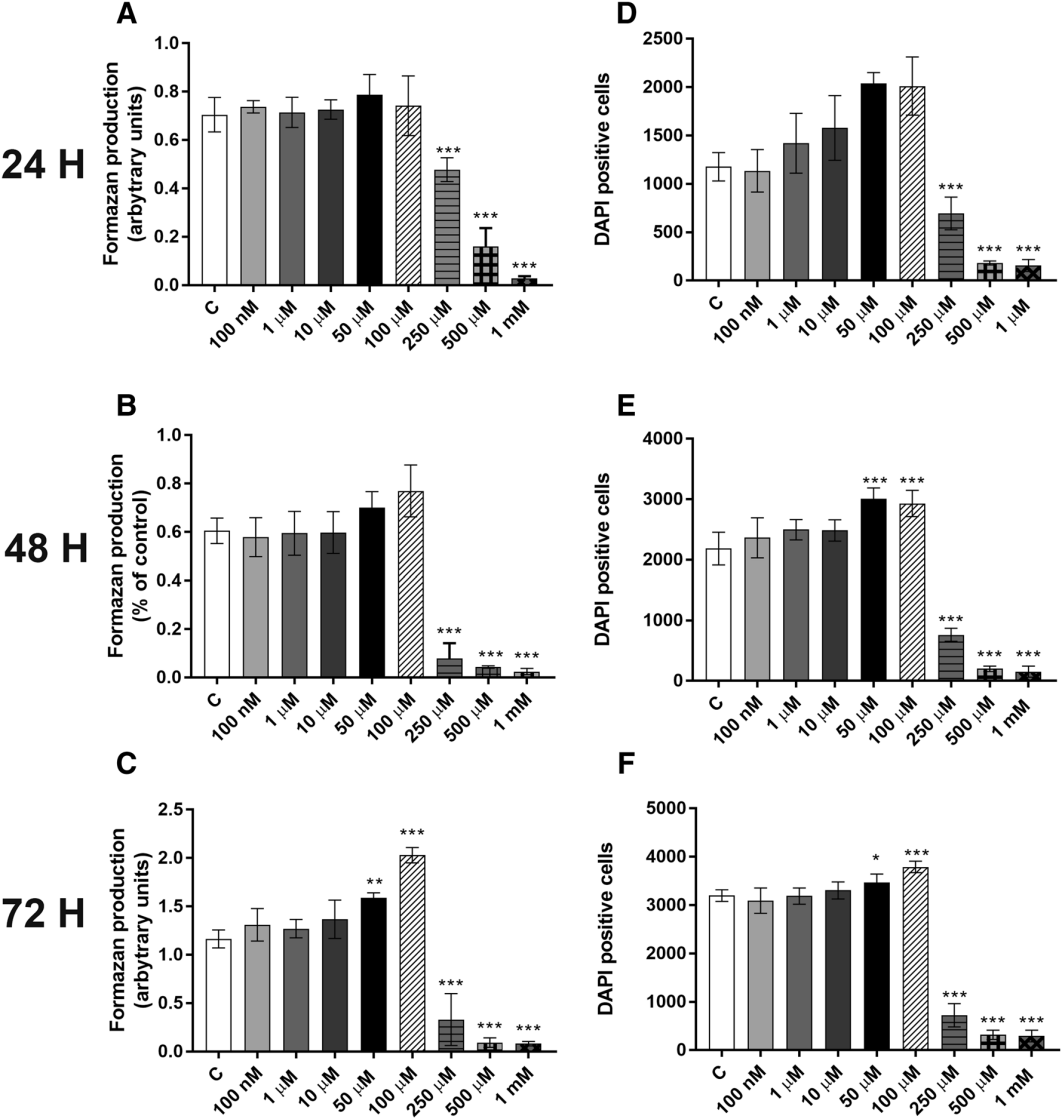
Effects of PFOA on the viability of MCF-10A cells. The cells were exposed to 0–1 mM PFOA for 24, 48 and 72 h. The viability was determined by MTT assay (**a**–**c**) and DAPI staining (**d**–**f**). Values represent mean ± SD from three independent experiments. Statistically significant differences from control are indicated as follows: ***p < 0.001 and *p < 0.05 (one-way ANOVA followed by the Tukey–Kramer test)

We first studied the effects of PFOA exposure on MCF-10A viability. Cells were incubated with 0–1 mM PFOA for 24, 48 and 72 h, and the cell viability determined by the MTT assay. The results showed that exposure to PFOA at 50 and 100 µM for 72 h increased the MTT production (Fig. 1c). In contrast, exposure to concentrations equals to 250 µM or higher decreased cell viability at all time points (Fig. 1a–c). To confirm these results, we determined the number of cells using DAPI staining. PFOA increased the number of cells at the concentrations of 50 and 100 µM at 48–72 h exposure (Fig. 1e, f), while the compound caused a decrease in the number of cells in the concentrations from 250 µM and higher at all time points (Fig. 1d–f).

### PFOA alters the cell cycle in MCF-10A cells

Since we found an increase in cell proliferation of cells treated with 50–100 µM PFOA, we determined the effects of PFOA exposure on cell cycle distribution using PI staining and flow cytometry analysis. Table 1 shows the mean percentage of gated cells for each DNA content fraction (*G*_0_/*G*_1_, *S, G*_2_/*M*) following 24, 48 and 72 h exposure to 100 µM PFOA. The results demonstrated that PFOA exposure significantly reduced the percentage of cells in *G*_0_/*G*_1_ phase and increased the percentage of cells in *S* phase at all time points.

**Table 1:** Effects of PFOA (100 µM) on MCF-10A cell cycle

	24 h			48 h			72 h		
	*G*_0_/*G*_1_	*S*	*G*_2_/*M*	*G*_0_/*G*_1_	*S*	*G*_2_/*M*	*G*_0_/*G*_1_	*S*	*G*_2_/*M*
Control	68.3 ± 15	17.0 ± 10	10 ± 6.17	51.3 ± 6.7	34.0 ± 6.8	14.7 ± 0.3	56.0 ± 5.4	33.1 ± 7.9	10.9 ± 2.6
PFOA	47.7 ± 4.7***	37.2 ± 4.1**	15 ± 1	33.3 ± 2.4**	58.5 ± 4.4**	8.1 ± 2.8**	33.6 ± 2.13**	60.6 ± 2***	5.6 ± 0.7***

Results as percentage of total events (10,000 events). Statistically significant differences from control are indicated as follows: ***p < 0.001 and **p < 0.01 (Student’s *t* test)

### The levels of proteins involved in cell cycle regulation are altered by PFOA

**Fig. 2:**
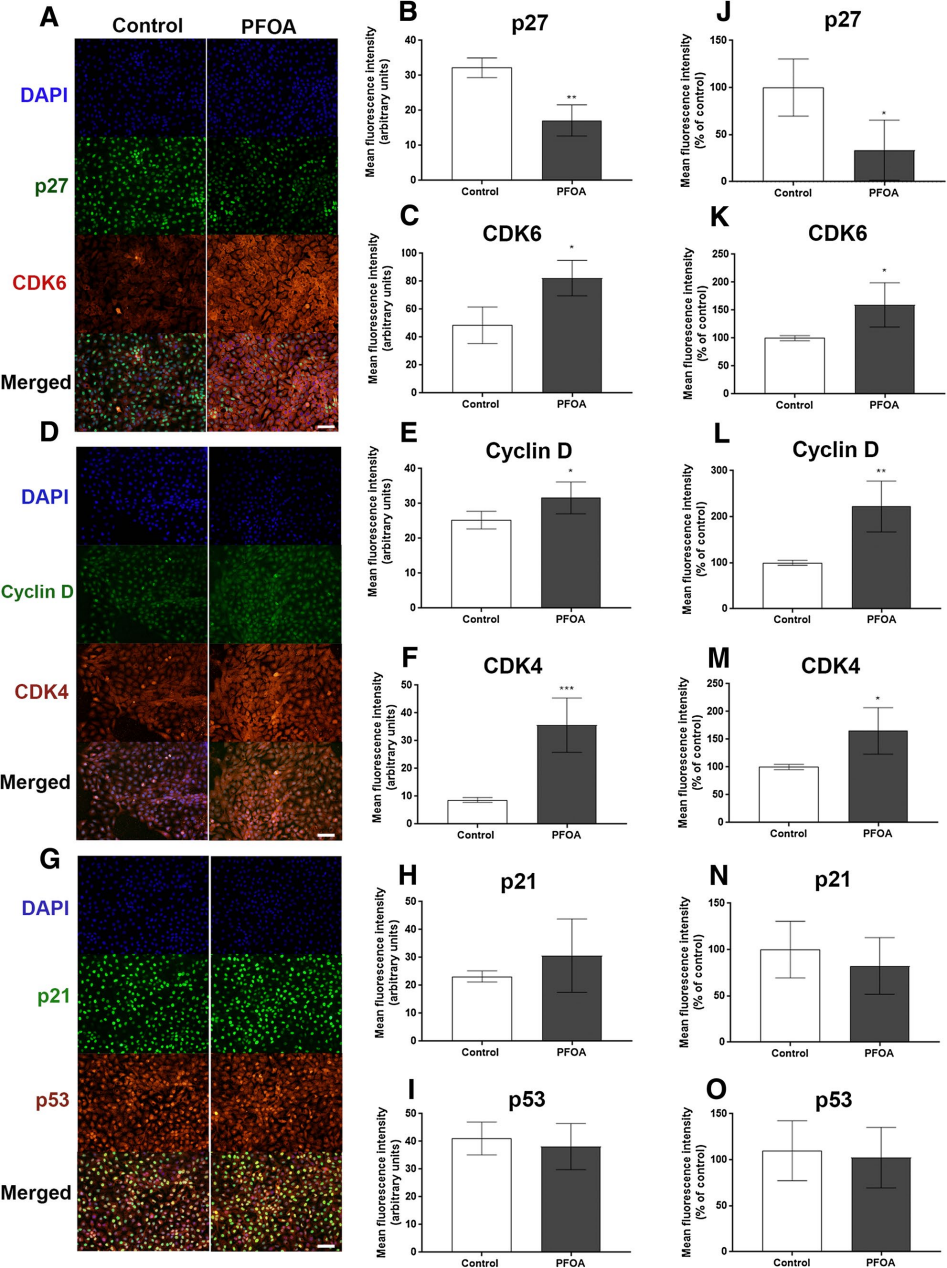
Effects of PFOA on the levels of proteins involved in cell cycle regulation. The cells were exposed to 100 µM PFOA for 72 h before immunocytochemistry and flow cytometry was performed. Representative images of PFOA-treated cells immunostained with p27 and CDK6 (**a**), cyclin D1 and CDK4 (**b**) and p21 and p53 (**c**). Mean fluorescence intensity was analyzed with immunocytochemistry (**b**–**i**) and flow cytometry (**j**–**o**) as described in “Material and methods” section. Values represent mean ± SD from three independent experiments. Scale bar = 50 µm. Statistically significant differences from control are indicated as follows: ***p < 0.001; **p < 0.01 and *p < 0.05 (Student’s *t* test)

To investigate the mechanisms involved in PFOA-induced cell proliferation and the alteration of the cell cycle in MCF-10A cells, the levels of the cyclin-dependent kinases (CDKs) CDK4, CDK6, cyclin D1 and their respective inhibitors (p27, p21 and p53) were analyzed by immunocytochemistry and flow cytometry. The fluorescence microscopy images revealed a reduced p27 level (Fig. 2a, b) and increased CDK6 (Fig. 2a, c), CDK4 and cyclin D levels (Fig. 2d–m), with no alteration on p21 and p53 levels (Fig. 2g–o). Confirming these results, flow cytometry analysis showed a decrease in the mean fluorescence intensity in p27-staining (Fig. 2j), and an increase in the fluorescence intensity in CDK6, CDK4 and cyclin D staining (Figure 2k–m) in PFOA-treated cells compared to the control group.

### PFOA exposure stimulates MCF-10A migration and invasion

**Fig. 3:**
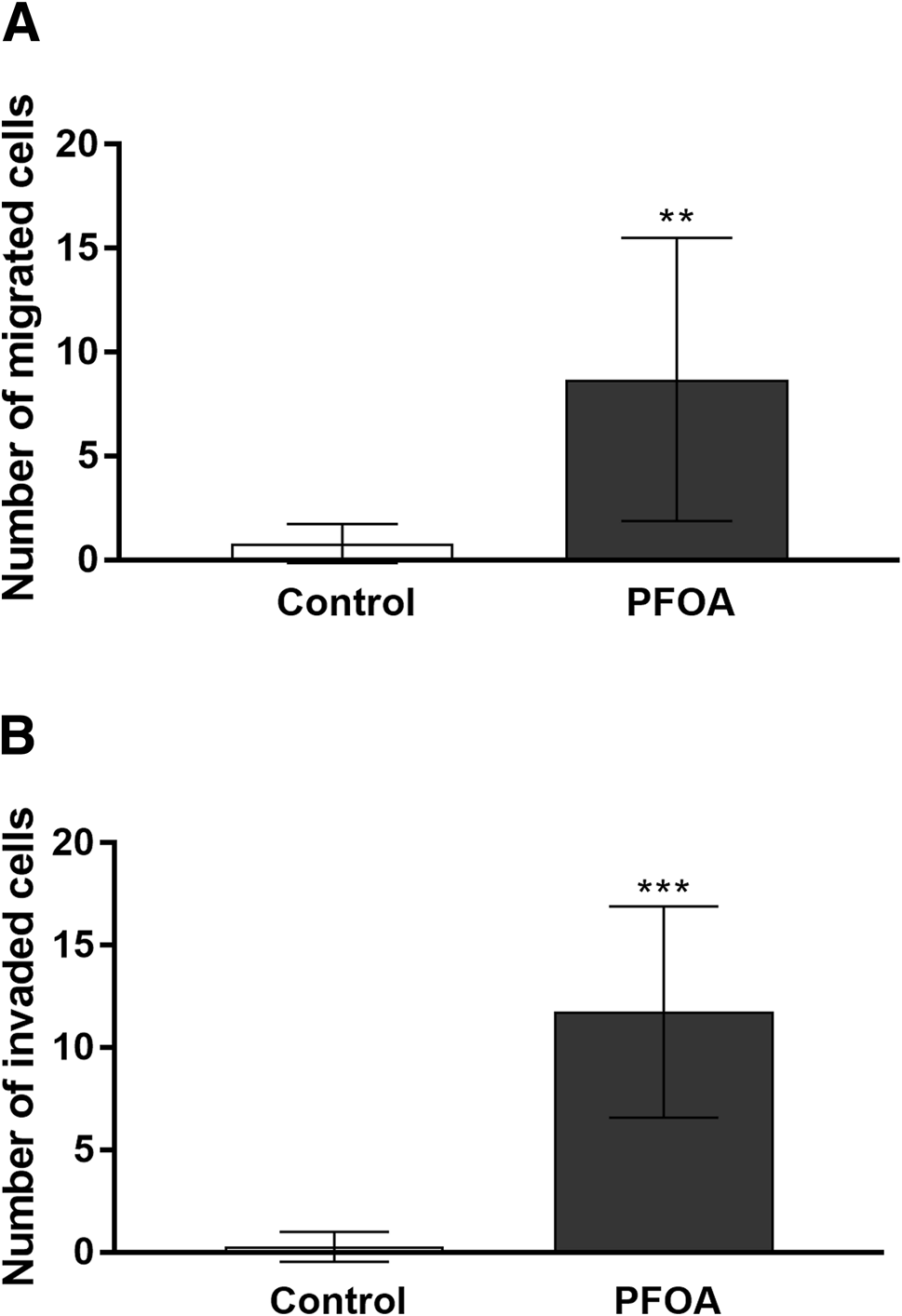
Effects of PFOA on MCF-10A cell migration and invasion capacity. Effects of PFOA on MCF-10A cell migration (**a**) and cell invasion (**b**) by a transwell assay. Migrated or invaded cells in the bottom were fixed with 4% formaldehyde and stained with DAPI and counted as described in the “Material and methods” section. Values represent mean ± SD. Statistically significant differences from control are indicated as follows: ***p < 0.001 and **p < 0.01 (Student’s *t* test)

To examine the involvement of PFOA on cell aggression, we performed a transwell migration and matrigel invasion assays. PFOA treatment at 100 µM significantly promoted cell migration and invasion of MCF-10A cells (Fig. 3a, b), suggesting that PFOA can induce MCF-10A transformation.

### Effects of PFOA on ER levels

**Fig. 4:**
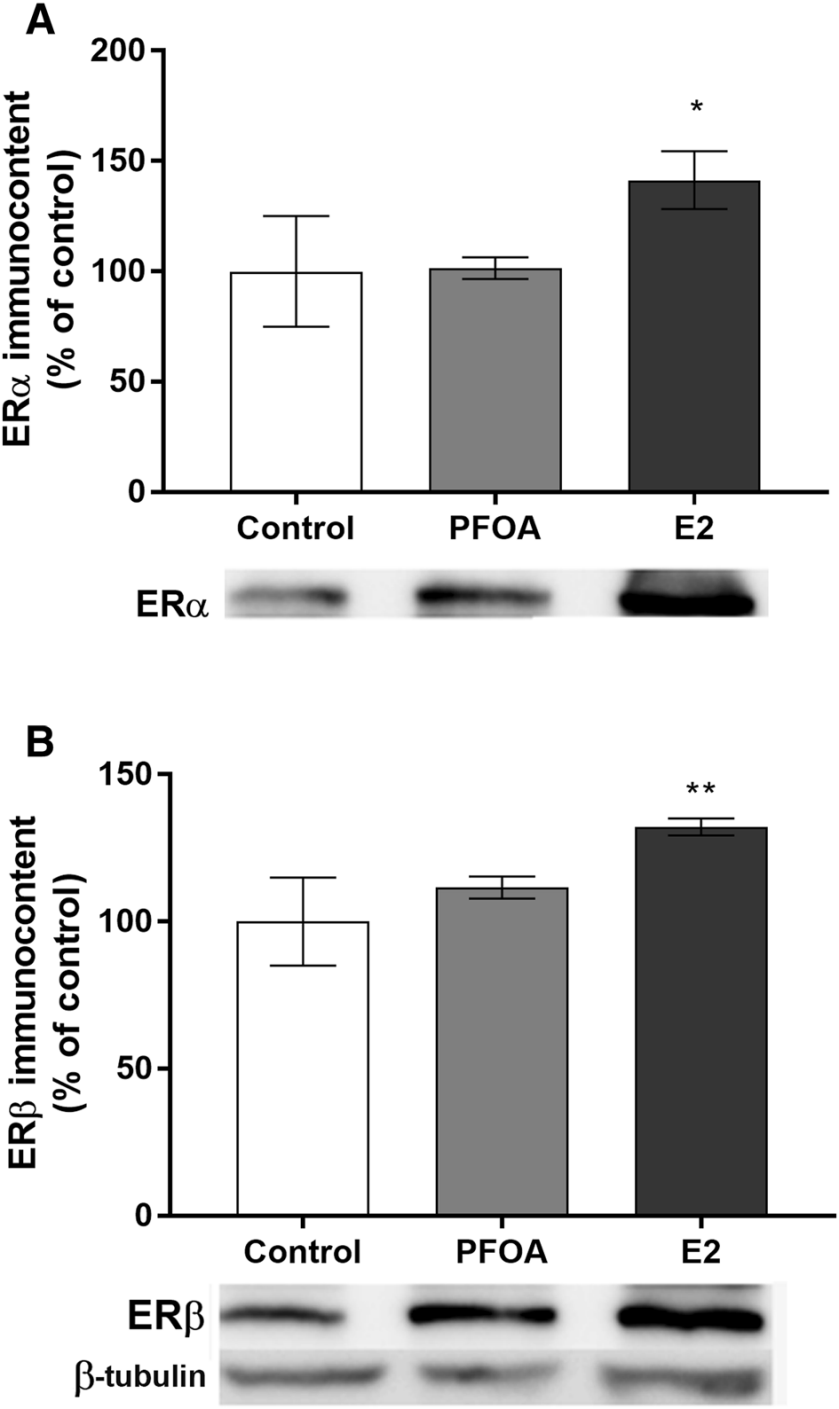
Effect of PFOA and 17β-estradiol (E2—positive control) on ERα (**a**) and ERβ (**b**) protein levels in MCF-10A breast cells. The cells were exposed to 100 µM PFOA or 10 nM E2 for 72 h. β-tubulin was used as a loading control. Representative blots of three experiments are shown. The results of densitometry analysis are expressed as ER protein band density normalized to the density of β-tubulin bands. Data are reported as mean ± SD of three independent experiments. Statistically significant differences from control are indicated as follows: **p < 0.01 and *p < 0.05 (one-way ANOVA followed by the Tukey–Kramer test)

Since previous studies have reported that PFOA has estrogenic effects (White et al. 2011; Kjeldsen and Bonefeld-Jorgensen 2013), and that MCF-10A cells can be transformed into a malignant phenotype by estrogenic compounds (Hemachandra et al. 2012), we investigated the PFOA effects on ER levels. Western blot analysis with ERα and β antibodies and 17β-estradiol (E2) as a positive control was performed. E2 was found to increase both ERα and ERβ levels after 72 h of exposure, while PFOA had no effects on ER levels (Fig. 4a, b).

### Hormone receptors involvement in the effects caused by PFOA on MCF-10A proliferation

**Fig. 5:**
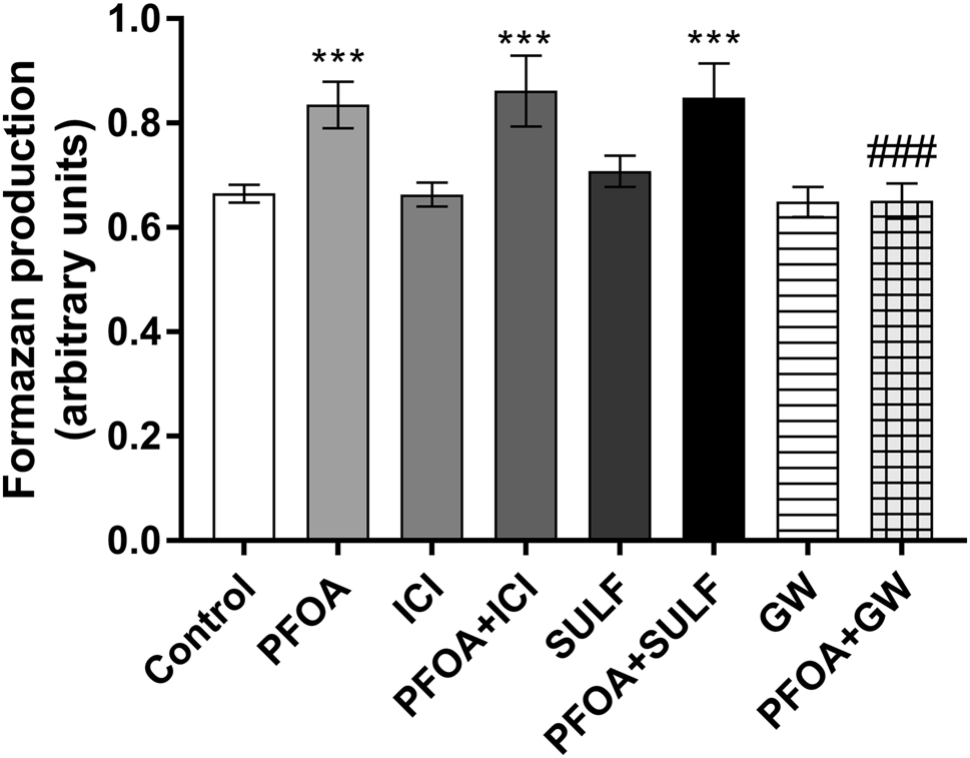
Involvement of endocrine receptors in the effects induced by PFOA. To determine the role of endocrine receptor, activation cells were preincubated with 100 nM ICI 182,780, 1 µM sulforaphane or 1 µM GW 6471 followed by 100 µM PFOA and the viability was determined by the MTT assay. Data are reported as mean ± SD. Statistically significant differences are indicated as follows: ***p < 0.001 compared with control and ^###^*p* < 0.001 compared with PFOA group (two-way ANOVA followed by the Tukey–Kramer test)

The ER blocker ICI 182,780 was used to examine the role of ER activation on PFOA-induced proliferation. However, the treatment of MCF-10A with ER blocker did not prevent the stimulatory effects of PFOA on cell proliferation (Fig. 5). Since one suggested mode of action for PFOA-induced toxicity is agonism of the nuclear hormone receptor PPAR-α, and activation of the PXR also may occur (Biegel et al. 2001; Lau et al. 2007), we then investigated the involvement of these two receptors. The results showed that the PPARα blocker GW 6471, but not the PXR antagonist sulforaphane was able to prevent MCF-10A proliferation induced by PFOA (Fig. 5).

## Discussion

The breast cancer incidence has globally increased during the last decades (Jemal et al. 2011). Risk factors and mechanistic pathways that can lead to mammary carcinogenesis need to be further elucidated. Recent experimental and epidemiological research suggest an association of the ubiquitously detected environmental contaminants, PFOS and PFOA with tumorigenesis, including breast cancer (Bonefeld-Jorgensen et al. 2011; Lau et al. 2007; Vieira et al. 2013; Wielsoe et al. 2017). However, the causative molecular and biochemical events that may lead to the adverse health outcomes after exposure to these PFAAs are largely undefined.

We recently reported that PFOS promotes MCF-10A proliferation by altering important regulatory cell cycle proteins and accelerating the cell cycle (Pierozan and Karlsson 2018). Despite the structural and many functional similarities, PFOS and PFOA have different toxicological effects (Tsuda 2016). Corroborating with this, the present study revealed that PFOA induces proliferation and acceleration of cell cycle by affecting different regulatory cell cycle proteins. PFOA-induced MCF-10A proliferation by down-regulation of p27 and up-regulation of CDK6, CDK4 and cyclin D levels. Similar to PFOS, PFOA-induced cell migration and invasion, illustrating their capability to induce neoplastic transformation of human normal breast epithelial cells. Unlike PFOS, the ER was not involved in the effects caused by PFOA in MCF-10A cells.

A characteristic feature of malignant tumors is deregulation of the cell cycle, which can occur on many levels, including one or several of the proteins involved in cell cycle control and progression. The progression through the *G*_1_–*S* phase—an important step in tumor development—is regulated by changes in the activity of specific CDKs, with CDK2/CDK4-CDK6 controlling the transition from *G*_1_ to *S* phase (Lundberg and Weinberg 1998). These enzymes, along with their corresponding cyclins D, allow the cell to enter or not in the *S* phase (Berthet and Kaldis 2007). During *G*_1_ phase, the predominant cyclin-CDK complexes are cyclin D-CDK4/6. Here, we found that PFOA increase cyclin D as well as CDK4/6 levels. This could explain the increase in MCF-10A proliferation. Cyclin D is overexpressed in up to 50% of primary breast cancers (Sutherland and Musgrove 2004). Interestingly, an association between overexpression of the cyclin D gene and hormone receptor expression has been observed in breast tumors (Jares et al. 1997; Worsley et al. 1996). These observations support the hypothesis that one of the mechanisms by which endocrine disruptors stimulates breast cancer cell proliferation might be through cyclin D induction.

In addition, CDK4/6 has a pivotal role in the *G*_1_–S-phase cell cycle transition in cancer (O’Leary et al. 2016). CDK4 is also found to be highly expressed in aggressive tumors and its expression correlate with poor overall and relapse free survival outcomes as well as poor prognostic features of breast cancer patients, suggesting a central role for this protein in cancer development and progression (Massague 2004). Moreover, up-regulated CDK6 is associated with the development of several types of cancers, and its high expression confers resistant of treatment in breast cancer cells (Alves et al. 2016; Tadesse et al. 2015).

Simultaneous with the increase in cyclin D and CDK4/6 levels, we found a decrease in the levels of the CDK inhibitor p27. Because of their inhibitory activity on cell cycle progression, the inhibitors are considered potential tumor suppressor genes. p27 negatively regulates the *G*_1_–*S* phase progression by binding to and inhibiting cyclin E-CDK2 or cyclin D-CDK4 (Sherr and Roberts 1999). p27 is one of the more recently highlighted cell cycle-related proteins and has the potential to predict outcome in several types of tumors. This protein effectively induces cell cycle arrest and decreases cyclin-CDK activity in breast cancer cell lines (Craig et al. 1997). Low levels, or loss, of p27 expression is a significant predictor of reduced survival, tumor progression and prognosis (Abbas and Dutta 2009; Catzavelos et al. 1997). Moreover, the p27 levels usually decreases during tumor development and progression (Vidal and Koff 2000), and there is considerable evidence that p27 inactivation is fundamental for the development of malignancies (Loda et al. 1997).

Metastasis is the leading cause of cancer-related death in most tumor types. Invasive cancers may spread into surrounding tissue, enter local vasculature, and metastasis to distant sites (McAllister et al. 2011). Cancer cell invasion involves the breaching of tissue barriers by cancer cells, and the subsequent infiltration of these cells throughout the surrounding tissue (McSherry et al. 2007). The first barrier faced by invasive cancer cells is the basement membrane, a dense and rigid matrix. Using a transwell and matrigel assay, we showed that PFOA enhanced the migration and invasion capacity of MCF-10A cells. This effect could be related to the decreased levels of p27, since it is well known that decreased concentration of this inhibitor is implicated in high tumor grade and metastasis in several tumor types including breast carcinomas (Alkarain and Slingerland 2004).

Most of the human breast cancers are initially estrogen dependent (Black et al. 1983). Estrogen-activated ERα regulates the expression of several key cell cycle regulatory genes, such as c-myc, c-fos and cyclin D1 (Prall et al. 1997; van der Burg et al. 1989). On the other hand, studies have reported that ERβ is frequently lost during carcinogenesis, suggesting a role for ERβ as a tumor suppressor (Park et al. 2003; Skliris et al. 2003). In the present study, PFOA had no effect on ERα and ERβ protein expression in MCF-10A cells. Moreover, in contrast to PFOS, the ER blocker ICI 182,780 was not able to prevent the cell proliferation caused by the compound. Instead, the results revealed that the PFOA stimulatory effects on MCF-10A proliferation involve activation of PPARα.

PFOA has previously been shown to activate PPARα, which may be the primary identifiable mode of action for PFOA-induced toxicity (Wolf et al. 2008). PPARα is a transcriptional regulator of lipid and glucose metabolism but also important for diverse functions as keratinocyte differentiation and skin diseases including epidermal and melanoma tumors (Michalik and Wahli 2007; Yang et al. 2006b). Although limited research has investigated the possible link between PPARα and breast cancer, the biology of the gene suggests that it could play a role in the pathology. One compelling hypothesis involves the PPARα-dependent modulation of cell cycle regulatory genes and effects on cell proliferation. According with this, mice fed with the potent PPARα ligand Wy-14 643 show an up-regulation of mRNA and protein for cyclin D1 and CDK4, which agree with our findings. In addition, a transgenic mouse study has suggested that altered PPARα-signaling has the potential to greatly interfere with proper differentiation of the mammary gland (Yang et al. 2006a), which is in line with the finding that PFOA exposure can cause a significant reduction in mammary differentiation in dams (White et al. 2007).

**Fig. 6:**
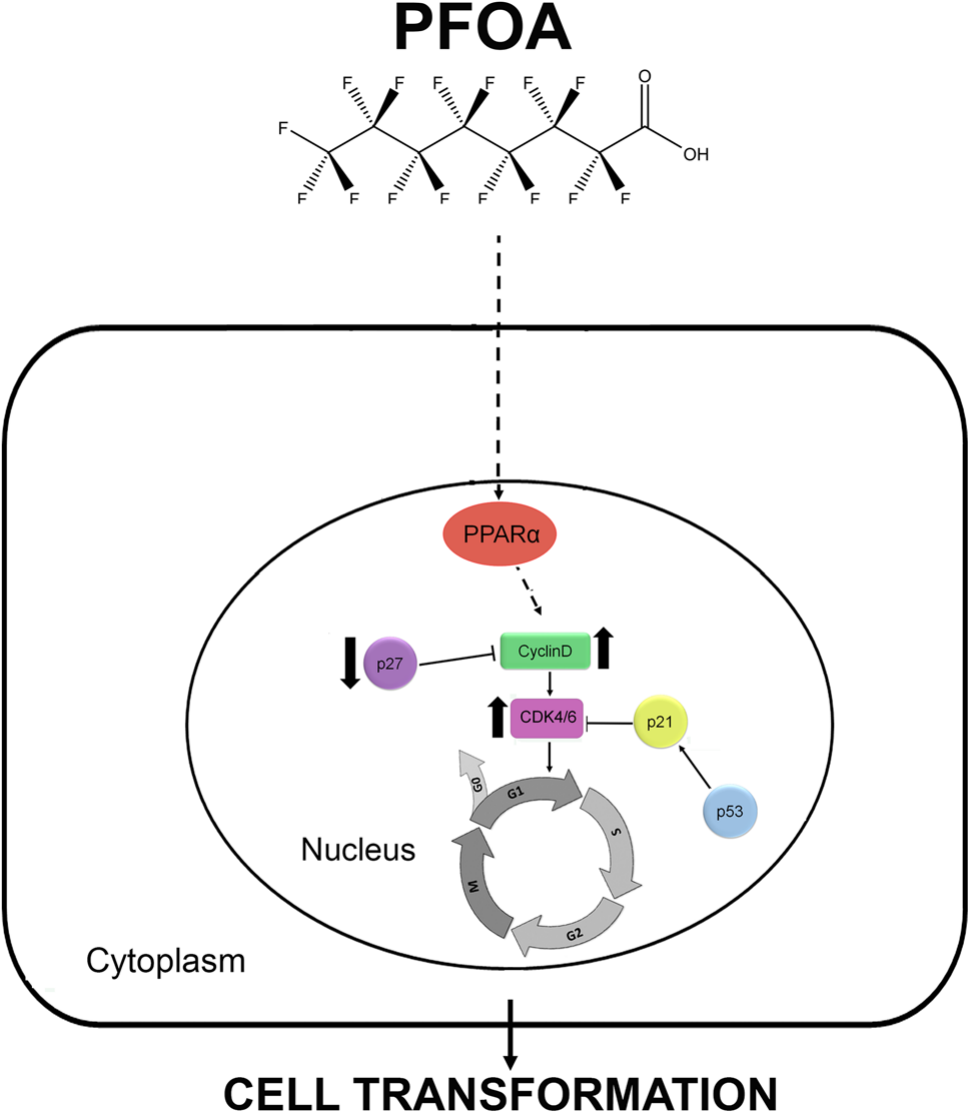
Schematic model of how PFOA may promote cell cycle progression, stimulate cell proliferation and transformation in MCF-10A cells. PFOA acts through PPARα, up-regulating cyclin D1 and CDK4/6 and down-regulating p27, to drive cells into the cell cycle from *G*_1_ to *S*, and promote cell cycle progression

Taken together, PFOA promotes proliferation of MCF-10A cells, induces cell cycle progression by up-regulating the levels of cyclin D1, CDK4/6 and down-regulating the CDK inhibitor p27, through non-estrogenic mechanisms. The compound also increases the metastatic potential of MCF-10A cells by inducing the capability of migration and invasion. This indicates that PFOA is capable of transforming the human normal breast epithelial cell line MCF-10A to a malignant profile. Blocking of PPARα prevented the cell proliferation, suggesting a vital role of this receptor. An overview of the proposed mechanism of PFOA toxicity is depicted in Fig. 6. The detailed mechanisms responsible for the effects of PFOA is unclear, and further studies are needed to shed light on the *in vivo* effect of PFOA on breast cancer development.
